# Association Between Behavioral Phenotypes and Paid Subscription and Renewal Among New mHealth App Users: Six-Month Prospective Cohort Study

**DOI:** 10.2196/93691

**Published:** 2026-09-10

**Authors:** Youssef Genaidy, Roshan Hasan, Julia Incitti, Erika L Bloom, Paul Tremblay, Marc S Mitchell

**Affiliations:** 1 Western University, 1151 Richmond Street, London, ON, N6A 3K7, Canada, 1 519-661-2111 ext 87936; 2 WayBetter Inc, Wilmington, DE, United States

**Keywords:** digital health, telemedicine, latent class analysis, health behavior, cluster analysis

## Abstract

**Background:**

Mobile health (mHealth) app effectiveness may be limited by low engagement. Increasing understanding of factors influencing engagement may help. Paid mHealth app subscription and renewal are two engagement metrics of particular interest to commercial app developers.

**Objective:**

This study aims to identify homogeneous user subgroups (ie, behavioral phenotypes) within a paid mHealth app context and examine associations with app subscription and renewal.

**Methods:**

A 6-month prospective cohort study was conducted with new users of the WayBetter (WayBetter Inc.) app, a subscription-based mHealth app. Through convenience sampling, users initiating a 7-day free trial between November 2023 and January 2024 were recruited. Latent class analysis (LCA) using the 3-step Bolck-Croon-Hagenaars (BCH) approach derived distinct classes, or phenotypes, from 33 indicator variables spanning three areas: (1) purpose-built survey responses (eg, sociodemographics), (2) device-assessed health data (eg, daily step count), and (3) 7-day free trial period engagement data (eg, app opens). Candidate LCA models ranging from 1 to 7 classes were generated. Model fit was assessed using several fit indices (eg, Vuong-Lo-Mendell Rubin likelihood ratio test [VLMR test]) alongside theoretical interpretability. Phenotypes were named based on distinct indicator probability profiles. Logistic regression was then used to estimate odds ratios (ORs) comparing the likelihood of subscription and renewal among users assigned to their most likely class, relative to a reference phenotype.

**Results:**

The sample included 934 users (mean age=41.53 [SD 9.65] years, 91.97% women [859/934], 72.16% with a chronic disease diagnosis [674/934]). Based on LCA fit indices (eg, VLMR test=467.16, *P*=.03), the 5-class LCA model was selected: (1) high trial period engagers, (2) moderate trial period engagers with multimorbidity, (3) moderate trial period engagers with lower disease burden, (4) low trial period engagers with multimorbidity, and (5) low trial period engagers with lower disease burden. Chronic disease diagnosis and total app opens emerged as the strongest drivers of class distinction. Phenotypes 1‐3 had greater odds of subscribing (OR 21.31, 95% CI 8.56-53.06; OR 7.11, 95% CI 4.04-12.50; OR 8.28, 95% CI 4.26-16.08, respectively) than phenotype 4 (OR 0.82, 95% CI 0.48-1.41), compared to phenotype 5 (the reference). Additionally, renewal odds for phenotypes 1‐4 were 1.06 (95% CI 0.62-1.81), 0.90 (95% CI 0.54-1.49), 0.99 (95% CI 0.58-1.69), and 0.93 (95% CI 0.48-1.80), respectively (vs reference).

**Conclusions:**

This study extends mHealth engagement research by identifying behavioral phenotypes within a sample of subscription-based app users. Using early survey, device-assessed health, and app engagement data, five phenotypes were identified. Notably, two phenotypes were at risk of low engagement (ie, nonsubscription). Although these five phenotypes may be less useful for predicting 6-month renewal, they could support early identification of users unlikely to subscribe by the end of the free trial period, possibly allowing for more timely, targeted onboarding strategies to reduce dropout and increase the likelihood of longer-term healthy habit formation.

## Introduction

### Problem

With an estimated 4.8 billion smartphone users globally [1], smartphone-based interventions (eg, via mobile health [mHealth] apps) have become a focal point for decision makers seeking to promote health behaviors [2-5]. Meta-analyses of randomized controlled trials investigating the efficacy of mHealth apps suggest they can stimulate small improvements in the short term (<6 months) [6]. Low mHealth app engagement, however, may limit intervention effect sizes and longer-term (>6 months) impact [7,8]. Evidence suggests most app users, for example, start to disengage shortly after initial download [9], and roughly 70% of users delete mHealth apps within the first hundred days [10]. In addition, evolving mHealth app business models have led to the rise of more paid mHealth apps that rely on subscriptions and subscription renewals (ie, increasingly important mHealth app engagement measures) [11] to stabilize or grow business revenue and drive intervention sustainability and longer-term health behavior.

Interest in boosting mHealth app engagement has led to greater integration of theoretical concepts in their design [12]. Behavioral economics (BE), an offshoot of traditional economics complemented by insights from psychology [13], may offer practical solutions for the persistent mHealth app engagement problem. Briefly, BE describes how systematic errors in human decision-making, known as “decision biases,” can result in poor health-related choices [14]. Among other things, BE suggests financial incentives (FI), such as paying people to eat better or walk more, may encourage health behavior adoption by exploiting the human tendency to act in favor of one’s immediate self-interest [15]. Evidence from meta-analyses suggests FI-based interventions can stimulate multiple health behaviors in a variety of populations across different social and cultural contexts [16-18]. FI can be prohibitively costly, however, when delivered on a population-scale [19]. The popular Canadian FI-based mHealth app, Carrot Rewards (Carrot Insights Inc), went out of business in 2019 in large part because of the cost of offering FI (about US $0.04 per user per day) indefinitely to its over one million users [19,20].

Broader application of BE concepts could drive incentive costs down and increase scale potential [21]. “Loss aversion” is a BE concept that describes how people are more motivated to change behavior to avoid a potential loss compared with receiving an equal gain [22]. This has inspired clever FI designs that involve potential losses, such as self-funded “deposit contracts,” where individuals wager their own money which they can earn back contingent on behavior [23]. In addition to possibly amplifying incentive effects, deposit contracts introduce (partial) cost-sharing by recipients as well. The same aversion to losses believed to increase effectiveness, however, may also deter people from entering deposit contracts in the first place. The limited evidence to date suggests low voluntary deposit contract engagement (ie,<15% uptake in randomized settings) [24] with very little known about contextual factors influencing engagement (eg, population and program characteristics) [24-27]. Further research into the factors influencing deposit contract-based mHealth app engagement may help refine this promising approach.

### Review of Relevant Scholarship

mHealth app engagement has been shown to vary with sociodemographics (eg, gender) [28,29], psychological characteristics (eg, personality traits) [30], and individuals’ current and past health behaviors (eg, physical activity) [29]. For instance, in a systematic review of 17 studies using mHealth apps for chronic disease self-management, younger age, high health literacy, and prior mHealth app use were shown to positively influence engagement [31]. Another review found those who scored higher in neuroticism, agreeableness, and openness on the big 5 personality traits were more likely to engage with a smartphone-based health intervention [32]. In addition, early mHealth app engagement metrics (eg, number of app opens, time spent on the app) have also been shown to independently predict future engagement [33,34]. Very little is known, however, about the interactive effects of all these potential engagement moderators when studied together. For example, while high levels of neuroticism have been linked to poorer health behaviors, this trait can help increase health behavior adherence when co-occurring with high levels of conscientiousness [30]. Several statistical approaches can help explore the dynamic interactions of variables moderating mHealth app engagement. Among the techniques available, latent class analysis (LCA) has demonstrated superiority over other classification techniques like multidimensional scaling and cluster analysis [35-37]. LCA uses a statistical modeling approach that uses model goodness-of-fit indices to determine the probability of individuals belonging to each class of a latent categorical variable [35]. LCA, in this instance, may help identify distinct homogeneous subgroups of mHealth app users (ie, “latent classes,” or behavioral phenotypes) at risk of low app engagement based on shared patterns in observed variables (ie, “latent class indicators”) [29,38].

### Hypotheses, Aims, and Objectives

The overarching aim of this study is to improve understanding of homogeneous user subgroups (ie, behavioral phenotypes) within a paid mHealth app context. Specifically, the primary objectives of this study are to (1) identify behavioral phenotypes among WayBetter (WayBetter Inc.) app users and (2) examine associations between phenotypes and paid subscription and 6-month subscription renewal. We hypothesize that groups of users characterized by co-occurring traits, such as risk-taking, poorer health, and higher app engagement, will emerge and differ in their odds of paid subscription and subscription renewal compared to a reference group.

## Methods

### Conditions and Design

This was a 6-month prospective cohort study that used LCA. No study conditions were experimentally manipulated; participants were observed under naturalistic conditions while using a commercially available deposit contract-based mHealth app. This study was reported in accordance with the Journal Article Reporting Standards (JARS) [39].

### Sampling Procedures

WayBetter Inc. is a US-based mHealth app company that has had over 1.5 million paying users in the company’s history across their suite of apps, most of whom are US residents. Their flagship app, the WayBetter app, was released in 2020. At the time this study was conducted, downloading the app was free of charge, but users had to pay a 6-month subscription (ie, US $69) to access key app features beyond the 7-day free trial period, most notably deposit contract-based WayBetter games. Games target lifestyle health behaviors (ie, physical activity, dietary habits, and mindfulness) and are offered in a group format (no limit on the number of players; hundreds of players per game is common) over a range of durations (eg, 2–⁠12 week) and deposit amounts (called an “investment” or “financial commitment” in the app; eg, US $5-$50 per game). The objective is for players to achieve the game’s health behavior goal (eg, 5000 steps per day on 4 days per week, tracking daily water consumption on 6 days per week). Players verify their activities by submitting photo or video evidence or connecting a fitness tracker (eg, step count), as applicable for the game. Players who do not achieve the game’s goal forfeit their deposit. Players achieving the game’s goal are declared winners and split the total “pot” of deposits, receiving a full refund of their deposit plus an equal share of the forfeited deposits (eg, a winner may earn their US $5 back plus US $1 profit; Multimedia Appendix 1 for app screenshots and detailed feature mapping). The app is primarily marketed to individuals pursuing weight loss. All new WayBetter users (ie, 18 years or older) starting a 7-day free trial between November 2, 2023 and December 19, 2023 (Regular season users) as well as December 26, 2023 and January 3, 2024 (High season users; defined as the postholiday period when enrollment historically increases) were invited via email to participate. Participants were recruited using a convenience sampling approach, whereby all eligible new users starting a 7-day trial during the recruitment windows were invited by email on day 5 of their respective 7-day free trial. Even if the user had already disengaged (stopped using the app) and/or canceled their free trial prior to day 5, they still received the email and had the option to participate.

### Inclusion and Exclusion

Eligible participants were new WayBetter users aged 18 years or older who initiated a 7-day free trial during the study recruitment periods. Users were excluded from the analytic sample if they had incomplete online survey responses (ie, started but did not finish or submit survey; only a fully completed survey could be submitted), had created a WayBetter account before the recruitment period, or deleted their account before 7-day free trial period app engagement data could be extracted. Key indicator variable data needed for phenotype estimation were missing among those with unsubmitted surveys and deleted accounts. Multiple imputation was deemed not suitable in these instances as entire indicator variable sources (eg, online survey and trial period engagement) were missing, and thus there was insufficient observed information on which to base reliable imputation [40].

### Participant Characteristics

The online survey collected sociodemographic characteristics, including age, gender identity, household income, and living classification, as well as psychological characteristics, chronic disease status, and previous health behaviors, including physical activity (Multimedia Appendix 2).

### Sample Size, Power, and Precision

The achieved analytic sample of 934 users was considered adequate based on previous LCA research showing that samples of this size can support accurate class enumeration [36].

### Measures and Covariates

A clear rationale for the inclusion of all indicator variables in LCAs is important given that observed indicators determine phenotype characteristics [36]. In this study, 33 indicator variables across three areas were selected: (1) online survey items, (2) device-assessed health behavior, and (3) 7-day trial period engagement data. First, a 27-item survey developed by Hasan [41] collected data in three domains: sociodemographics (eg, gender and income), psychological characteristics (eg, physical activity self-efficacy, conscientiousness, and risk-taking), and health behaviors (eg, fruit and vegetable intake and weight loss attempts). This survey was systematically developed using an evidence-based approach [42,43] which included choosing indicators from preestablished survey categories in similar studies, selecting survey items most likely to be associated with mHealth engagement, and ensuring survey items align with at least one aspect of the Capability, Opportunity, and Motivation Leading to Behavior (COM-B) model (ie, a framework explaining behavior as resulting from the interaction of capability, opportunity, and motivation) [44] (Multimedia Appendix 3). Indicator selection also considered the deposit contract-based WayBetter context, particularly regarding risk-taking and weight loss. Aligning survey items with conceptual links to the COM-B model allowed for a more structured interpretation of how individual characteristics (eg, chronic disease diagnosis) may act as potential barriers or facilitators of mHealth engagement [44]. Additionally, linking survey items to the three factors from COM-B known to generate behavior change (ie, capability, opportunity, and motivation [44]) can support more targeted intervention recommendations (eg, a user’s psychological capability may be boosted through interventions targeting self-efficacy). Second, device-assessed health behavior data, namely body weight and average daily step count, were collected during app sign-up. Lastly, rather than including all WayBetter app engagement metrics (eg, game type and deposit size), 7-day trial period engagement data (eg, app opens and time spent on app) previously shown to predict WayBetter engagement [34] were included (Multimedia Appendix 4 contains a full description of the LCA indicator variable selection process as well as the full list of engagement variables included and operational definitions). As well, because the aim of this study was to identify early behavioral phenotypes in a deposit contract-based mHealth app, we prioritized indicators that reflected broader onboarding and engagement patterns rather than metrics tied only to WayBetter’s specific game mechanics, such as game type. Notably, and like other mHealth engagement studies using LCA [29,45], some of the included indicator variables may conceptually overlap. These were retained, however, because they were deemed to capture distinct, theoretically relevant dimensions of user health status and engagement. For example, specific conditions such as depression, anxiety, and obesity were distinguished from overall chronic disease burden, while total app opens, later trial period opens, and total seconds on the app captured related but nonidentical dimensions of early engagement.

### Data Collection

Upon providing informed consent, participants completed an online survey including sociodemographic, psychological characteristic, and previous health behavior questions (Multimedia Appendix 2). Device-assessed health behavior (eg, daily step count) and engagement data (eg, app opens) during the 7-day trial were collected as well. Survey, health behavior, and 7-day trial engagement data were used to form behavioral phenotypes. Six months of engagement data (eg, games joined) were collected for users subscribing after the trial period. The distal engagement outcomes of interest were paid subscription after the 7-day trial along with whether their subscription was renewed at 6 months. Both outcomes were coded as binary variables (ie, yes or no).

### Data Diagnostics

Among eligible participants, survey responses “Prefer not to answer” and “I do not know” were coded as missing data, as were any missing device-assessed or engagement data (via unexpected technology glitches, for example). Little’s Missing Completely At Random (MCAR) test was conducted to assess whether missing indicator data were consistent with MCAR. Missing indicator values were handled using maximum-likelihood missing-data estimation via Mplus software (Muthén & Muthén; version 8.10), allowing participants with partially observed indicator data to contribute to model estimation.

### Analytic Strategy

LCA is a latent variable modeling technique that aims to identify the optimal number of homogeneous subgroups in a population, known as “latent classes,” using a set of two or more observed indicator variables [46,47]. LCA enables the identification of unobserved subgroups, such that groups are distinct and the probability of an individual’s membership can be estimated [38]. Mplus software and the 3-step Bolck-Croon-Hagenaars (BCH) approach were used to execute the LCA [48-50], in part because the 3-step BCH approach enables class-specific regression onto distal outcomes [51].

In the first BCH step, the LCA was run using the 33 indicator variables and sequentially generated seven classes. The model of best fit was identified using several fit indices (ie, Akaike information criterion [AIC], adjusted Bayesian information criterion [aBIC], Vuong-Lo-Mendell Rubin likelihood ratio test [VLMR test], the bootstrap likelihood ratio test [BLRT], and entropy; Multimedia Appendix 5). In step 2, using posterior probabilities, individuals were assigned to their most likely class and corresponding measurement errors were estimated. In step 3, the latent class model from step 1 was adjusted to account for the measurement error estimated in step 2. Distal engagement outcomes (ie, app subscription and subscription renewal) were regressed onto latent class membership using the 3-step BCH approach. Renewal status (ie, yes or no) was assessed only among users subscribing after the trial period, because users not subscribing were not eligible to renew. Notably, classes were named based on distinct indicator probability profiles (ie, patterns of conditional response probabilities across indicator variables), rather than on the distal outcomes of interest. Indicators were considered distinguishing if there was a ≥50% difference in conditional probabilities between classes (stronger drivers) or a ≥30% difference (weaker drivers). Subscription and renewal were not included as indicator variables in the LCA. Additional details regarding the BCH procedure have been provided in the Multimedia Appendix 5.

Finally, exploratory post hoc binary logistic regression was conducted using a sample subset (ie, subscribers only) to better understand predictors of subscription renewal. Variables driving class distinction in the LCA, along with whether a game was joined during months 1‐6, were included in the regression to examine their potential associations with renewal. Games joined is a WayBetter*-*specific metric previously shown to drive engagement [34].

### Bias

Potential sources of bias included the convenience sampling method and recruitment on day 5 of a 7-day trial. Self-selection into survey participation inherently creates bias towards users who may be more engaged with the app (ie, had not disengaged or canceled their free trial prior to day 5). As a result, the analytic sample likely overrepresents app users who were still engaged late in their free trial period. Additionally, participants with incomplete survey responses or deleted accounts before data extraction were excluded which may limit the generalizability of the identified phenotypes to all new WayBetter users.

### Ethical Considerations

Ethical approval for this study was obtained from the Western University Human Research Ethics Board (No. 123207). All participants provided informed consent before completing the online survey. To protect participant privacy and mitigate conflicts of interest related to industry partner participation, only Western University research team members had access to survey responses and conducted analyses examining the relationship between survey responses and WayBetter app-use metrics. Participant email addresses were collected to verify WayBetter account eligibility and distribute the study compensation. Before analysis, direct identifiers were removed from the analytic dataset, and participants were represented using study identification codes only. Participants who completed the survey were compensated with a US $10 Amazon (Amazon.com, Inc) gift card. No images in the paper or additional files contain personal identifiers for participants of the study.

## Results

### Participant Flow

**Figure 1.**
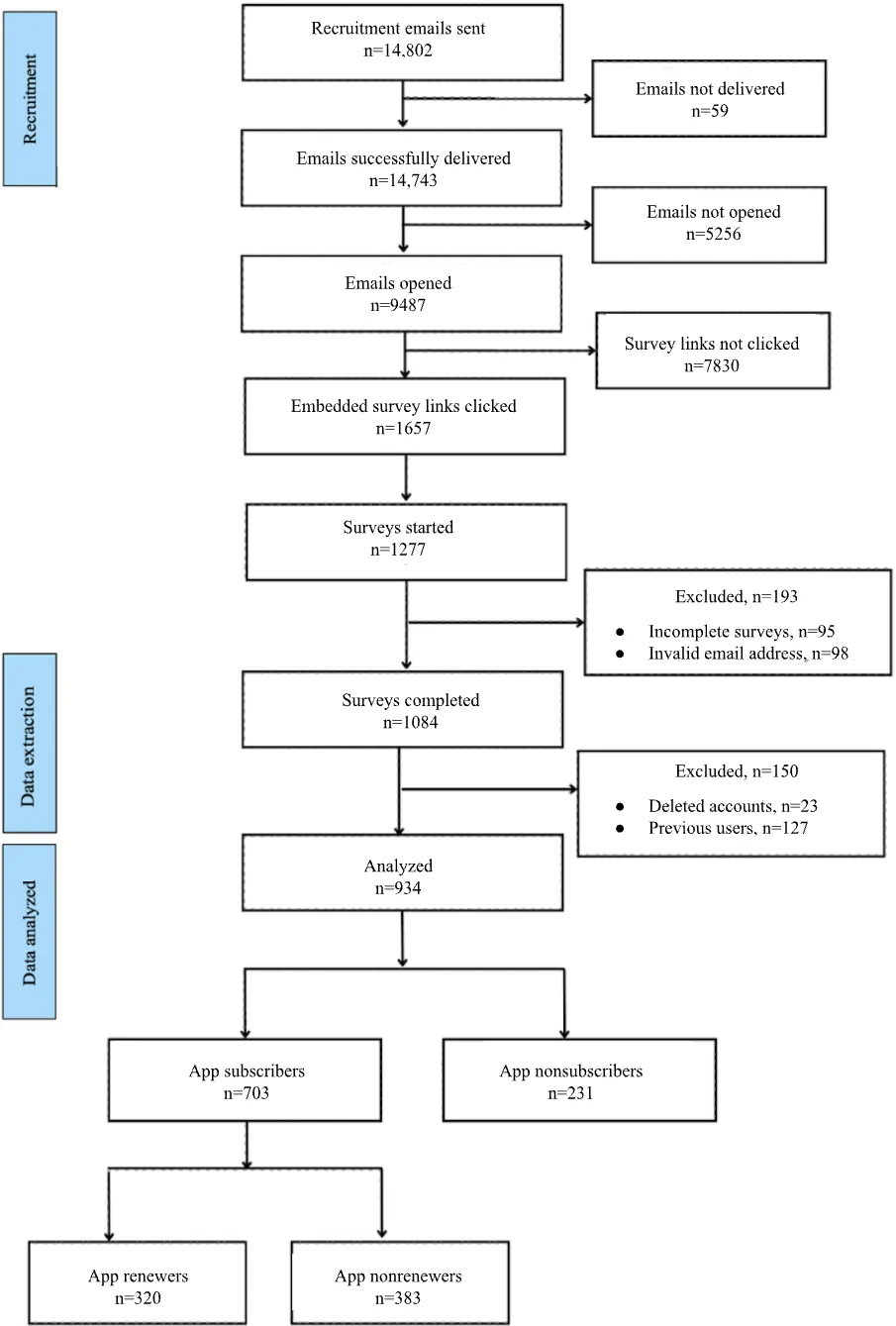
Participant flowchart for the 6-month prospective cohort study of new WayBetter app users initiating a 7-day free trial. Incomplete survey responses: 1 or more missing answers. Deleted accounts: users who completed the survey but deleted their WayBetter account prior to the data extraction date. Previous users: users who had created a WayBetter account prior to the study recruitment period and were not initiating their first WayBetter trial.

A total of 14,802 study recruitment emails were sent to new WayBetter users. Of these, 1084 users completed the online survey (about 7% recruitment rate), as illustrated in Figure 1. Participants were subsequently excluded if they had deleted their account (n=23) or were identified as previous WayBetter users (n=127), resulting in a final analytic sample size of 934 users.

### Recruitment

Recruitment occurred from November 2, 2023, to December 19, 2023 (regular season), and from December 26, 2023, to January 3, 2024 (high season) via email invitations.

### Statistics and Data Analysis

At least 1 indicator was missing for 56 (roughly 6%, 56/934) participants (ie, “Prefer not to answer” or “I do not know” survey responses; and inaccessible device-derived data). The Little MCAR test was statistically significant, (*χ*²_797_=873.22; *P*=.03) indicating evidence against data being MCAR. Missingness pattern inspection suggested that missing data were concentrated within a small number of variables, particularly sensitive survey questions (ie, household income and weight history) as well as app or device-derived variables (ie, step count and self-reported weight).

About 13% (124/934) of participants self-reported an annual household income of less than $50,000 (based on their country’s currency), and 15% (143/934) reported living in a rural area (Table 1). Nearly half (about 48%; 448/934) indicated living with two or more chronic diseases, and about 80% (736/934) of study participants reported physical activity below guideline levels [52]. Descriptive statistics suggest baseline characteristics for regular and high season users were similar with some minor differences (eg, high season users reported slightly higher household income and a greater prevalence of chronic disease compared to regular users).

**Table 1.** Sample characteristics of new WayBetter app users stratified by regular season and high season recruitment periods.^a^

Characteristics	Regular season, n=408	High season, n=526	Total sample, n=934
Age (years), mean (SD)	41.36 (9.84)	41.68 (9.49)	41.53 (9.65)
Gender identity, n (%)			
Man	32 (7.84)	31 (5.89)	63 (6.75)
Woman	369 (90.44)	490 (93.16)	859 (91.97)
Gender-diverse	2 (0.49)	1 (0.19)	3 (0.32)
Nonbinary	4 (0.98)	4 (0.76)	8 (0.86)
Prefer to self-describe	0 (0)	0 (0)	0 (0)
Prefer not to answer	1 (0.25)	0 (0)	1 (0.11)
Annual household income, n (%)			
Less than $29,999	28 (6.86)	22 (4.18)	50 (5.35)
$30,000–$49,999	35 (8.58)	39 (7.41)	74 (7.92)
$50,000–$69,999	70 (17.16)	69 (13.12)	139 (14.88)
$70,000–$89,999	41 (10.05)	73 (13.88)	114 (12.21)
$90,000–$109,999	61 (14.95)	63 (11.98)	124 (13.28)
$110,000+	149 (36.52)	237 (45.06)	386 (41.33)
I do not know	3 (0.74)	5 (0.95)	8 (0.86)
Prefer not to answer	21 (5.15)	18 (3.42)	39 (4.18)
Living classification, n (%)			
Large city	74 (18.14)	81 (15.40)	155 (16.60)
Suburb near a large city	172 (42.16)	234 (44.49)	406 (43.47)
City or small town	100 (24.51)	129 (24.52)	229 (24.52)
Rural area	62 (15.20)	81 (15.40)	143 (15.31)
Prefer not to answer	0 (0)	1 (0.19)	1 (0.11)
Number of chronic diseases, n (%)			
None	109 (26.72)	151 (28.71)	260 (27.84)
One	86 (21.08)	140 (26.62)	226 (24.20)
Two or more	213 (52.21)	235 (44.68)	448 (47.97)
Physical activity, n (%)			
75 minutes or less	179 (43.87)	270 (51.33)	449 (48.07)
75–150 minutes	128 (31.37)	159 (30.23)	287 (30.73)
≥150 minutes	98 (24.02)	93 (17.68)	191 (20.45)
Prefer not to answer	3 (0.74)	4 (0.76)	7 (0.75)

a Income is based on the participant’s country currency. Chronic diseases are conditions diagnosed by a health professional that are expected to last or have already lasted six months or more (ie, depression, arthritis, asthma, obesity, etc). Physical activity was measured as the number of moderate-to-vigorous physical activity minutes participants accumulated each week. Regular season users downloaded the app from November 2, 2023, to December 19, 2023. High season users from December 26, 2023, to January 3, 2024. Age was calculated using all participants with available baseline age data prior to exclusions (n=1084).

LCA models including up to 7 classes were tested. Considering model fit indices, the distribution of the sample across classes, and theoretical interpretability, a five-class LCA model was selected. The conditional probability weights for selected indicator response categories across the 5 classes are presented in Table 2. Although the four and five-class LCA models were similar on fit indices (Multimedia Appendix 6), further inspection of the five-class model revealed a distinct group of highly engaged users not found in the four-class model. Classification accuracy was high, with fit indices shown in Multimedia Appendix 6. Additionally, the AIC and aBIC were plotted to determine inflection points where the curve plateaus and the rate of improvement slows, meaning more classes do not yield the same decreases in values of AIC and aBIC [36]. Odds ratios (ORs) were used to compare the likelihood of subscription and renewal for each class or phenotype, using phenotype 5 as the reference. In general, phenotype distinction was driven by chronic disease status and 7-day free trial period engagement.

**Table 2.** Conditional probability weights (as percentages) of selected indicator variables across the 5 latent classes.^a^

Indicator variable	Class 1 (n=166)	Class 2 (n=235)	Class 3 (n=169)	Class 4 (n=139)	Class 5 (n=225)
Depression, probability (%)					
No	61.60	49.30	100^b^	28.90	93.50
Yes	38.40	50.70	0	71.10^c^	6.50
Anxiety or panic disorder, probability (%)					
No	69.20	36.30	93	24.10	87.40
Yes	30.80	63.70	7	75.90^c^	12.60
Obesity, probability (%)					
No	59.30	38.70	90.90^b^	46.50	81.30
Yes	40.70	61.30	9.10	53.50^e^	18.70
Number of chronic diseases, probability (%)					
None	19.40	0	69.70^d^	0	48.30
One	31.40	7.50	30.30	0	49.30
Two or more	49.20	92.50^b^	0	100^c^	2.40
Total app opens, probability (%)					
0–7	0	0.40	0	97.20	80.10
8–11	0	42.10	27.80	2.80	19.90
12–15	0	40.70	36.40	0	0
16–25	44.30	16.80	35.80	0	0
26–66	55.70^d^	0	0	0	0
Total app opens day 4, probability (%)					
0–1	5.70	47.40	37.60	89.50	88
2–13	94.30^b^	52.60	62.40	10.50	12
Total app opens day 5, probability (%)					
0–1	6.90	40.80	36.90	91.80	90.40
2–21	93.10^b^	59.20	63.10	8.20	9.60
Total app opens day 6, probability (%)					
0–1	4.90	48.20	41.90	89.20	83.40
2–15	95.10^b^	51.80	58.10	10.80	16.60
Total app opens day 7, probability (%)					
0–1	8.10	43.80	40	92.80	91.20
2–17	91.90^b^	56.20	60	7.20	8.80
Total seconds on app, probability (%)					
0–2896	0	11.50	7.70	67.30	68.10
2897–4839	0	30.40	24.10	25.90	19.60
4840–10,706	18.90	49.50	52.50	6.10	11.70
10,707–93,187	81.10^d^	8.60	15.80	0.70	0.50

a Values represent the probability of each indicator response category within each latent class. For example, 71.10% for “Depression: Yes” in class 4 indicates that users assigned to class 4 had a 71.10% probability of reporting depression. Class 1: high trial engagers. Class 2: moderate trial engagers with multimorbidity. Class 3: moderate trial engagers with lower disease burden. Class 4: low trial engagers with multimorbidity. Class 5: low trial engagers with lower disease burden. Arthritis, asthma, depression, anxiety, or panic disorder are chronic diseases. Obesity is a chronic disease that is measured as BMI >30 kg/m2. Chronic diseases are conditions diagnosed by a health professional that are expected to last or have already lasted 6 months or more. Total app opens and total seconds on app, calculated during the 7-day free trial period.

b Indicates ≥30% deviation between subscribing classes.

c Indicates ≥50% deviation between nonsubscribing classes.

d Indicates ≥50% deviation between subscribing classes.

e Indicates ≥30% deviation or more between nonsubscribing classes.

### Phenotype 1: High Trial Period Engagers

Phenotype 1 made up 17.78% (166/934) of the total sample. The odds a user in phenotype 1 subscribed and renewed were 21.31 (95% CI 8.56-53.06) and 1.06 (95% CI 0.62-1.81) times higher, respectively, than for a user in phenotype 5 (Table 3). Total app opens and total time spent (stronger drivers) drove phenotype distinction. Notably, all users in this phenotype spent more than 80 minutes using the app and opened the app at least 16 times or more during the trial period, findings specific to phenotype 1. Total app opens on days 4‐7 were weaker drivers of phenotype 1 distinction.

**Table 3.** Associations between latent class membership and paid subscription after a 7-day free trial and 6-month subscription renewal among new WayBetter app users.

Outcome	Class 1 OR^a^ (95% CI)	Class 2 OR (95% CI)	Class 3 OR (95% CI)	Class 4 OR (95% CI)	Class 5^b^
Subscription	21.31 (8.56-53.06)	7.11 (4.04-12.50)	8.28 (4.26-16.08)	0.82 (0.48-1.41)	—^c^
6-month subscription renewal	1.06 (0.62-1.81)	0.90 (0.54-1.49)	0.99 (0.58-1.69)	0.93 (0.48-1.80)	—

a OR: odds ratio.

b Indicates reference class.

c NA: not applicable.

### Phenotype 2: Moderate Trial Period Engagers With Multimorbidity

Phenotype 2 made up 25.16% (235/934) of the sample. The odds a user in phenotype 2 subscribed and renewed were 7.11 (95% CI 4.04-12.50) and 0.90 (95% CI 0.54-1.49), respectively (vs reference group, phenotype 5). The health profile of users in phenotype 2, however, differed from phenotype 1. Specifically, number of chronic diseases was a weaker driver of phenotype distinction. There were no stronger drivers.

### Phenotype 3: Moderate Trial Period Engagers With Lower Disease Burden

Phenotype 3 made up 18.09% (169/934) of the sample. The odds a user in phenotype 3 subscribed and renewed were 8.28 (95% CI 4.26-16.08) and 0.99 (95% CI 0.58-1.69), respectively (vs reference group, phenotype 5). Unlike phenotypes 1 and 2, the health profile of individuals in phenotype 3 was characterized by lower chronic disease burden, driving phenotype distinction (stronger driver). Depression and obesity (weaker drivers) further drove phenotype distinction.

### Phenotype 4: Low Trial Period Engagers With Multimorbidity

Phenotype 4 made up 14.88% (139/934) of the sample. The odds a user in phenotype 4 subscribed and renewed were 0.82 (95% CI 0.48-1.41) and 0.93 (95% CI 0.48-1.80), respectively (vs reference group, phenotype 5). All users in this class reported living with two or more chronic diseases, driving class distinction (stronger driver). Depression, obesity, and anxiety or panic disorder were also stronger drivers.

### Phenotype 5: Low Trial Period Engagers With Lower Disease Burden

Phenotype 5 was the reference class and made up 24.09% (225/934) of the sample.

The results of the post hoc binary logistic regression revealed two variables associated with renewal among subscribers. Joining a game in months 5 and 6 was associated with higher odds of renewal (OR 2.39, 95% CI 1.27-4.49; OR 2.05, 95% CI 1.04-4.04, respectively). Joining a game in months 1‐4 did not predict renewal (Table 4). Obesity, depression, and living with no chronic disease or more than two chronic diseases also did not significantly predict renewal. Overall classification accuracy was 60.20%, with the model correctly predicting that a user did not renew their membership 88.50% of the time. The model correctly predicted a user renewed their subscription 26.30% of the time (Multimedia Appendix 7). The model was better at classifying nonrenewals than renewals; however, its low sensitivity for true renewals suggests limited predictive utility overall. Although the Hosmer-Lemeshow *P* value indicates the model fits the data reasonably well, the low Nagelkerke *R*^2^ value of approximately 4.7% suggests the model explains only a small portion of the variance in renewal (Multimedia Appendix 8). In other words, the model provides limited insight into the factors that drive renewal and would likely benefit from additional predictors.

**Table 4.** Post hoc binary logistic regression model examining predictors of 6-month subscription renewal among WayBetter users who subscribed after the 7-day free trial.^a^

Variable	Coefficient (B)	SE	Wald *χ*^2^ (*df*)	*P* values	OR^b^	95% CI
Games joined M1	−0.349	0.190	3.374 (1)	.07	0.71	(0.47-1.02)
Games joined M2	0.181	0.217	0.696 (1)	.40	1.20	(0.78-1.83)
Games joined M3	0.172	0.229	0.564 (1)	.45	1.19	(0.76-1.86)
Games joined M4	−0.295	0.287	1.057 (1)	.30	0.75	(0.43-1.31)
Games joined M5	0.869	0.323	7.239 (1)	.007^c^	2.39	(1.27-4.49)
Games joined M6	0.717	0.346	4.292 (1)	.04^c^	2.05	(1.04-4.04)
Obesity	0.077	0.193	0.159 (1)	.69	1.08	(0.74-1.58)
Chronic disease	−0.012	0.231	0.003 (1)	.96	0.99	(0.63-1.55)
Chronic disease (2+)	−0.251	0.268	0.877 (1)	.35	0.78	(0.46-1.32)
Depression	0.155	0.217	0.510 (1)	.48	1.17	(0.76-1.79)
Constant	−0.216	0.165	1.714 (1)	.19	0.81	—^d^

a M1–M6 indicate months 1 through 6 of the subscription period.

b OR: odds ratio.

c *P*<.05 indicates statistical significance.

d N/A: not applicable.

## Discussion

### Support of Original Hypotheses

Findings partially supported our hypothesis. First, behavioral phenotypes characterized by co-occurring traits emerged, supporting our expectation that meaningful groups of users would be identified. Second, several phenotypes differed from the reference group in their odds of paid subscription. However, none of the behavioral phenotypes differed significantly from the reference group in their odds of 6-month subscription renewal.

Using online survey as well as device-assessed health and early (ie, 7-day free trial) engagement data, five phenotypes were identified from a fairly homogeneous sample of nearly 1000 new WayBetter users: (1) high trial period engagers, (2) moderate trial period engagers with multimorbidity, (3) moderate trial period engagers with lower disease burden, (4) low trial period engagers with multimorbidity, and (5) low trial period engagers with lower disease burden. Several LCA indicator variables distinguished the phenotypes, with chronic disease diagnosis and total app opens being the strongest drivers. Notably, two phenotypes at risk of low engagement were identified (ie, phenotypes 4‐5). The early identification of these categories of users with co-occurring traits may guide more strategic mHealth intervention design in the future [29,45] (eg, targeted communications regarding larger initial reward or payout opportunities). On the other hand, LCA did not predict renewal using survey, health, and 7-day trial period engagement data.

### Similarity of Results

The results of this study are consistent with previous LCA studies in this area. One study used LCA to identify behavioral phenotypes among 442 discharged hospital patients using smartphones and wearable devices to track physical activity [29]. Using surveys to measure physical activity levels, sociodemographics, and behavioral traits, four phenotypes with varying intervention responses were identified: (1) more agreeable and conscientious; (2) more active, social, and motivated; (3) more risk-taking and less supported; and (4) less active, social, and risk-taking [29]. Similar to the results of this paper, the authors identified two phenotypes at risk for low engagement, with a high likelihood of discontinued wearable device usage [29]. Another study used LCA to identify three behavioral phenotypes among 602 overweight or obese adults participating in a 24-week physical activity intervention [45]. They were (1) more extroverted and more motivated, (2) less active and less social, and (3) less motivated and at-risk [45]. While the methodology was similar to this paper, the authors focused on baseline characteristics and intervention response, rather than early engagement metrics in an mHealth app [45]. Findings were conceptually aligned in that both studies identified an at-risk phenotype characterized by low motivation and poor outcomes. Finally, in a larger LCA, authors aimed to describe and identify digital technology access and health use patterns among 13,993 adults in the United States [53]. Consistent with this paper’s findings, distinct phenotypes were identified that may benefit from targeted digital interventions [53]. Two of the ten identified behavioral phenotypes were likely to benefit from interventions that improve health tool access [53]. This study extends this work by showing that within-app engagement patterns, rather than access alone, can meaningfully distinguish users at risk for disengagement.

### Interpretation

That phenotypes strongly predicted subscription, but not renewal, is a pivotal finding likely reflecting two different transtheoretical model (TTM) stages of behavior change [54]. Existing mHealth literature suggests that early engagement may be sufficient to determine whether a user is willing to initiate a longer-term paid commitment (ie, initiation stage of change), as trial-period app use can reflect immediate interest, novelty, and perceived intervention value [55,56]. In contrast, renewing a subscription after 6 months likely represents the maintenance stage of change [54]. TTM suggests maintenance requires health behaviors to become more routine, internalized, and resilient to common barriers over time, rather than simply initiated with a short motivation burst, for example [54,57]. That is, while the present latent classes captured factors relevant to initial adoption, they may not have captured whether app use had become sufficiently habituated across the subscription period to justify continued financial commitment in the form of a renewal. To further investigate predictors of renewal, a post hoc binary regression was conducted with a sample subset (ie, subscribers only) revealing that number of games joined in months 5 and 6 was potentially associated with renewal. Investing in app features likely to promote game joining in months 5 and 6 may offer one potential avenue for improving renewal rates [58], although further investigation into the factors influencing renewal is needed (eg, prior app subscription patterns).

One strength of this 6-month LCA study is the large sample size (n=934), which was adequate for class enumeration, though we acknowledge represents only about 7% (934/14,802) of invited users. Another strength is the range of indicator variables used and the longer-term evaluation of largely unexplored (but commercially relevant) engagement metrics may inform future LCA investigations and benefit mHealth app engagement and effectiveness more broadly. However, some limitations should be considered when interpreting the results of this study. First, LCA assumes local independence between phenotypes, meaning that the observed indicators within each class are presumed to be uncorrelated [38]. Although model fit (eg, AIC and aBIC), classification quality (eg, entropy and posterior class membership probabilities), class size (ie, no class with less than 10% of the sample), and interpretability (ie, theoretically coherent class profiles) supported the 5-class solution, several chronic disease status and app engagement indicators were conceptually related; therefore, some class distinctions may partly reflect residual associations among correlated indicators rather than fully independent latent dimensions. Future studies should assess the stability of these phenotypes using more parsimonious indicator sets [37]. Second, Little’s MCAR test suggested missing data were not MCAR [59], with missingness concentrated in sensitive survey items and app or device-derived variables. Missing indicator data were handled using maximum-likelihood missing-data estimation in Mplus, rather than multiple imputation, allowing participants with partially observed survey data to contribute to the LCA while avoiding imputation of class-defining indicators that could have made phenotype estimation more dependent on the imputation model. Although this approach retained more of the analytic sample, the non-MCAR missingness pattern should be considered when interpreting the resulting phenotypes.

### Generalizability

The generalizability of these findings for the broader new WayBetter app user base may be limited due to the roughly 7% recruitment rate. The findings may be most applicable to new WayBetter users engaged through the latter portion of the 7-day free trial as well as those identifying as higher-income women living in suburban areas or small cities with one or more chronic diseases given how common these characteristics were in this sample. The timing and method of survey distribution may have introduced selection bias, with surveys distributed to users on day 5 of their 7-day free trial period. This likely resulted in more responses from individuals who were still engaged and/or had not yet canceled their free trial on day 5 compared to those who had already canceled their trial or disengaged by day 5. The phenotypes and associations reported here may have been different had all individuals downloading the app been included in analyses. This may have increased the observed prevalence of more engaged and subscription-prone phenotypes, and decreased the prevalence of early-disengaging phenotypes or those associated with a lower likelihood of subscription. In addition, the statistically significant MCAR test indicated that the missingness mechanism is inconsistent with MCAR, introducing the possibility that participants with missing indicator variable data differed from those with complete data. However, rejecting MCAR does not distinguish between missing at random (MAR) and missing not at random (MNAR) [60,61]. If the data were MNAR, phenotype estimation could have been biased, potentially limiting generalizability [61]. Phenotypes formed with this sample may also not be generalizable to mHealth apps not marketed to weight loss pursuing individuals nor those that do not use similar deposit contract designs. Furthermore, the study sample consisted of primarily women (about 91%, 859/934), individuals with a household income of more than $110,000 (in their country’s currency, but primarily reported in United States dollars [USD]; about 41%, 386/934), individuals living in a suburban area near a large city (about 43%, 406/934), and individuals living with more than two chronic diseases (about 47%, 448/934). This raises important questions regarding digital health equity [62]. Prior work suggests that access to, comfort with, and sustained use of digital health technologies may differ meaningfully across gender, socioeconomic, and other demographic groups [62]. As such, a sample with greater male representation or lower household income, for instance, may have clustered differently, potentially yielding alternative latent class structures and different predictors of subscription behavior [21,55]. Accordingly, replication of this latent class approach within more diverse samples is needed before broader generalizations can be reasonably made.

### Implications

This study has several practical implications. First, results suggest several chronic diseases, including depression, anxiety, and obesity, may be prevalent among WayBetter’s user base. As such, an opportunity exists for WayBetter to deploy more personalized app features that meet the needs of users living with these conditions (eg, condition-specific goal framing, adaptive reminders, and feedback that adjusts to users’ mental health status or weight-related barriers to engagement) [63]. Additionally, given WayBetter’s predominantly woman-identifying user base, the company could explore developing features tailored to women that lead to a more personalized app experience [64]. Providing a more comprehensive approach to health (ie, physical, mental, and reproductive health) may also position the company to compete with larger, female-oriented mHealth apps [64] such as Flo (Flo Health) and Willow (Willow Innovations In) [65,66]. As for engagement-related considerations, app opens and time spent on app during days 4‐7 of the 7-day free trial period may mark a critical period for WayBetter to boost initial subscription, with less certainty regarding whether these same early metrics translate to longer-term retention via subscription renewal [33]. Results from the LCA revealed observable differences in engagement patterns between phenotypes at risk of low engagement compared to those with higher engagement. These patterns were noticeable during the 7-day trial period, suggesting this is a critical window for intervention (Table 2) [33]. Strategies to boost user engagement, such as increasing reward size (ie, payout following a wager), may be most beneficial if delivered during this time frame for users with similar engagement patterns [67]. Lastly, the binary regression revealed that games joined in months 5 and 6 were predictors of renewal. Therefore, WayBetter may benefit from adjusting their user retention strategies, by increasing the frequency and variety of games for users who have not joined games, for example, in the months leading up to a user’s renewal date to counteract attrition [68]. However, because renewal ORs across latent classes were largely null and the post hoc renewal model explained only a small proportion of variance with limited sensitivity for true renewals, these findings should be interpreted as preliminary rather than definitive indicators of long-term retention.

While this study identified behavioral phenotypes using sociodemographic, health behavior, and trial period engagement data, it did not directly evaluate whether tailoring intervention components to these phenotypes has the potential to improve subscription, renewal, or longer-term health behavior change. Future work should therefore use experimental or quasi-experimental designs to assess whether early identification of at-risk phenotypes can inform targeted design features that meaningfully alter engagement trajectories [33,45]. Additional user-level data may strengthen future models. Although survey items were informed by prior studies, several did not predict subscription or renewal. Measures capturing long-term app intentions, their prior subscription patterns (eg, streaming services), and existing gym memberships, for example, may better explain engagement and retention in an mHealth context [69]. In addition, collecting survey data at multiple points during the user’s journey (eg, immediately after app download and before or after subscription) may improve prediction of longer-term engagement [55]. Incorporating more detailed, app-specific engagement metrics may further refine phenotype definitions [55]. Within WayBetter, variables such as game type, game duration, wager size, and win rate may provide deeper insight than broader metrics like app opens and time spent on the app. Finally, present findings regarding game participation in months 5 and 6 disproportionately predicting renewal suggest that renewal may be driven by late-stage engagement rather than early onboarding alone. Future interventions should therefore evaluate strategies aimed at sustaining engagement in the latter half of a subscription period [58] (eg, adaptive game recommendations, altered payout structures, and reengagement notifications prompted by declining participation). Testing these strategies within a randomized controlled trial may provide a more holistic understanding of the factors linking engagement, renewal, and sustained behavior change within deposit contract-based mHealth apps.

### Conclusion

mHealth apps hold great potential for promoting healthy behaviors at scale, but their impact ultimately depends on sustained user engagement. Despite a low recruitment rate, this study demonstrates that meaningful behavioral phenotypes can still be identified using sociodemographic, health, and early app engagement data. Importantly, these phenotypes showed clear utility in differentiating users who are more or less likely to subscribe following the free trial period. In contrast, prediction of 6-month renewal was considerably weaker, suggesting that longer-term retention may be explained by additional factors not captured in the present analysis. From both public health and commercial perspectives, the ability to identify mHealth users at risk for nonsubscription still offers practical value for improving initial onboarding and conversion to paid subscription. In the next phase of this research, the way in which behavioral phenotypes engage with mHealth apps will be examined. These analyses may help refine mHealth app design, ultimately helping target behavioral phenotypes that are less likely to engage with refined or brand-new intervention features in the future.

## Supplementary material

10.2196/93691 Multimedia Appendix 1 WayBetter app screenshots and key app features, behavior change techniques, and behavioral economic constructs.

10.2196/93691 Multimedia Appendix 2 Study survey instrument.

10.2196/93691 Multimedia Appendix 3 Survey item information.

10.2196/93691 Multimedia Appendix 4 Indicator variable selection rationale and operational definitions for engagement and device-assessed health metrics.

10.2196/93691 Multimedia Appendix 5 Latent class analysis model selection criteria and Bolck-Croon-Hagenaars procedure.

10.2196/93691 Multimedia Appendix 6 Model fit information and selection criteria for latent class models.

10.2196/93691 Multimedia Appendix 7 Post hoc logistic regression classification accuracy.

10.2196/93691 Multimedia Appendix 8 Post hoc logistic regression model fit statistics.

## Abbreviations

aBIC: adjusted Bayesian information criterion
AIC: Akaike information criterion
BCH: Bolck-Croon-Hagenaars
BE: behavioral economics
COM-B: Capability, Opportunity, and Motivation Leading to Behavior
FI: financial incentive
JARS: Journal Article Reporting Standards
LCA: latent class analysis
MAR: missing at random
MCAR: missing completely at random
mHealth: mobile health
MNAR: missing not at random
OR: odds ratio
TTM: transtheoretical model
USD: United States dollar
VLMR test: Vuong-Lo-Mendell Rubin likelihood ratio test
