# Supplementary material for: Association Between Behavioral Phenotypes and Paid Subscription and Renewal Among New mHealth App Users: Six-Month Prospective Cohort Study
Source: J Med Internet Res. 2026 Sep 10;28:e93691. doi: 10.2196/93691 (PMC13561440; doi:10.2196/93691)
Supplement: Supplementary file 1 [file jmir-v28-e93691-s001.docx]

WayBetter app screenshots and key app features, behavior change techniques, and behavioral economic constructs.

**Figure S1.** WayBetter app screenshots: (a) personal plan page, (b) 5K steps a day game entry screen, (c) and game list.

Table S1. Key WayBetter app features, behavior change techniques, and behavioral economic constructs.

| **Key Feature** | **Example** | **BCTs** | **BE Decision Biases** |
| --- | --- | --- | --- |
| 1. “***Games”****:* Users are encouraged to enter games targeting small lifestyle modifications aimed to incrementally build healthy habits. | “*5k Steps a day*” is a two-week game where users set a goal to reach 5000 steps four out of seven days each week. | 1.1, 1.4, 1.8, 8.3 | Present Bias  Salience Bias |
| 2. “***Bets”****:* Users can bet anywhere from $10–$100 USD of their *own money* on games. This is a *deposit contract.* | “*5k Steps a day*” requires users to bet $10 of their own money upfront, which they can earn back if the game is won. | 10.1, 10.7, 10.11 | Commitment  Loss aversion |
| 3. “**Invite loops”:** Users are encouraged to invite friends and family to join a game as a team. | “*5k Steps a day*” is offered in a partner format: “*5k Steps Partner Walk”* where both users must meet step count requirements to win. | 1.4, 3.1, 12.2, 13.1 | Herd Behavior |
| 4. “**Progress Tracking**”: Real-time progress displayed as graphs for goal visualization. | Users are encouraged to update weight (pounds) each week to track their progress towards a goal. | 1.1, 1.4, 1.8, 1.9 | Salience Bias |
| 5. **“My Plan”** Personalized plans that prompt users to take part in their health behavior goal setting. | Examples of personalization plan prompts included: *“confirm your goal”* and *“pick a motivational booster”.* | 1.1, 1.4, 1.8, 1.9 | Salience Bias Commitment |

***Note.*** BCT: behavior change technique taxonomy [1]. BE: behavioral economics. Present bias: preference for a payoff close to the present time rather than in the future. Loss aversion: the strong aversion to losses and the weaker desire for gains of equivalent magnitude. Herd behavior: people do what others are doing instead of making independent decisions. Salience: information that stands out, is novel, or seems relevant is more likely to affect our actions. Commitment: preference for a consistent self-image, best achieved by making a commitment or pre-commitment [2].

**References**

1.	Michie S, Richardson M, Johnston M, Abraham C, Francis J, Hardeman W, Eccles MP, Cane J, Wood CE. The Behavior Change Technique Taxonomy (v1) of 93 Hierarchically Clustered Techniques: Building an International Consensus for the Reporting of Behavior Change Interventions. ann behav med 2013 Aug;46(1):81–95. doi: 10.1007/s12160-013-9486-6

2.	Camerer CF, Loewenstein G. Advances in Behavioral Economics. Rabin M, editor. Princeton University Press; 2003. doi: 10.2307/j.ctvcm4j8jISBN:978-1-4008-2911-8
