# Supplementary material for: Association Between Behavioral Phenotypes and Paid Subscription and Renewal Among New mHealth App Users: Six-Month Prospective Cohort Study
Source: J Med Internet Res. 2026 Sep 10;28:e93691. doi: 10.2196/93691 (PMC13561440; doi:10.2196/93691)
Supplement: Supplementary file 2 [file jmir-v28-e93691-s002.docx]

**.** Study survey instrument.

There are 27 survey questions. This survey should take you about 5-10 minutes to complete. If you wish to skip a question, please select "prefer not to answer"

Please type your email here. You will receive a $10 Amazon gift card for completing this survey.

Your email MUST match your WayBetter account email to receive your gift card.

Text box: _____________

About You

1. What year were you born?

*Please enter your birth year.*

____________

2. What is your gender identity?
   - Man
   - Woman
   - Gender diverse
   - Non-binary
   - Prefer to self-describe (branch: text box)
   - Prefer not to answer
3. What is are your ethnic origins or ancestry? Select all geographic areas from which your ancestors first originated.

- Western Europe (eg, Greece, Sweden, United Kingdom)
- Eastern Europe (eg, Hungary, Poland, Russia)
- North Africa (eg, Egypt, Morocco, Sudan)
- Sub-Saharan Africa (eg, Kenya, Nigeria, South Africa
- West Asia / Middle East (eg, Iran, Israel, Saudi Arabia)
- South and Southeast Asia (eg, India, Indonesia, Singapore)
- East and Central Asia (eg, China, Japan, Uzbekistan
- Pacific / Oceania (eg, Australia, Fiji, Papa New Guinea
- North America (Canada, United States)
- Central America and Caribbean (eg, Jamaica, Mexico, Panama)
- South America (eg, Brazil, Chile, Colombia)
- Self-describe: _____ (branch -- Text box: Please self-describe your ethnic origins or ancestry)
- I don’t know
- Prefer not to answer

4. How would you identify yourself in terms of race? Select all groups that apply to you.

- Asian or Pacific Islander
- Black
- Indigenous (eg, North American Indian Navajo, South American Indian Quechua, Aboriginal or Torres Strait Islander)
- Middle Eastern or North African
- White
- Self-describe: _______ (branch -- Text box: Please self-describe how you identify in terms of race)
- I don’t know
- Prefer not to answer

5. Are you Hispanic or Latino/a/x?
   - Yes
   - No
   - Prefer not to answer
6. Which of the following best describes the place where you now live?

- A large city
- A suburb near a large city
- A small city or town
- Rural area
- Prefer not to answer

7. What was your total household income before taxes last year (in your country’s currency)? Select one:

*Please note: Income can come from various sources such as from work, investments, pensions or government. Examples of income include your pay, Employment Insurance, Social Assistance, Child Tax Benefit and other income such as child support, spousal support (alimony) and rental income. Additionally, ensure you include all members’ income of the household in the total.*

- Less than $29,999
- $30,000 – $49,999
- $50,000 – $69,999
- $70,000 – $89,999
- $90,000 – $109,999
- More than $110,000
- I do not know
- Prefer not to answer

8. Including yourself, how many family members live in your household?

- _______ (people)
- Do not know
- Prefer not to answer

9. Do you have any of the following chronic illnesses or conditions? These are conditions diagnosed by a health professional that are expected to last, or have already lasted, 6 months or more.

Arthritis (rheumatoid and osteoarthritis)

Osteoporosis

Asthma

COPD

Angina

Congestive heart failure or heart disease

Heart attack

Neurological disease

Stroke or transient ischemic attack

Diabetes type I or II

Peripheral vascular disease

Upper gastrointestinal disease

Depression

Anxiety or panic disorders

Visual impairment

Hearing impairment

Degenerative disk disease

Obesity and/or BMI of >30kg/m^2^

Another chronic illness not listed here

None

Prefer not to answer

About Your Personality

Please answer questions #9–11 using a scale from 1–10, 1 being not confident at all and 10 being completely confident:

10. How confident are you that you can be physically active even if you have to try several times until it works?
    - 1–10
11. How confident are you that you can stick to eating healthy foods even if you have to try several times until it works?
    - 1–10
12. How confident are you that you can lose weight even if you have to try several times until it works?

- 1–10

13. How do you see yourself? Are you generally a person who is fully prepared to take risks, or do you try to avoid taking risks?

Please use the scale to indicate response, where the value 0 means: 'not at all willing to take risks and the value 10 means: 'very willing to take risks'

- 0–10

14. “I see myself as dependable and self-disciplined”

*Please answer the extent to which the pair of traits applies to you, even if one characteristic applies more strongly than the other.*

- Disagree strongly
- Disagree moderately
- Disagree a little
- Neither agree nor disagree
- Agree a little
- Agree moderately
- Agree strongly
- Prefer not to answer

15. “I see myself as open to new experiences”

- Disagree strongly
- Disagree moderately
- Disagree a little
- Neither agree nor disagree
- Agree a little
- Agree moderately
- Agree strongly
- Prefer not to answer

16. “I believe that health and fitness apps are useful in my daily life”

- Strongly disagree
- Disagree
- Neutral
- Agree
- Strongly agree
- Prefer not to answer

17. “I have the knowledge necessary to use health and fitness apps”

- Strongly disagree
- Disagree
- Neutral
- Agree
- Strongly agree
- Prefer not to answer

18. “I find health and fitness apps easy to use”

- Strongly disagree
- Disagree
- Neutral
- Agree
- Strongly agree
- Prefer not to answer

19. “People whose opinion that I value prefer I use health and fitness apps”

- Strongly disagree
- Disagree
- Neutral
- Agree
- Strongly agree
- Prefer not to answer

20. “App features that make healthy living (eg, moving more, eating better) more joyful are important to me”

- Strongly disagree
- Disagree
- Neutral
- Agree
- Strongly agree
- Prefer not to answer

21. “App features that educate me about how to exercise best are important to me”

- Strongly disagree
- Disagree
- Neutral
- Agree
- Strongly agree
- Prefer not to answer

About Your Past

22. In general, how many servings of vegetables do you have in a day?

*(One serving = 1/2 cup of cooked vegetables, ½ cup beans, peas or lentils. Includes fresh, frozen and canned vegetables.)*

- 0
- 1
- 2
- 3
- 4
- 5 or more
- Prefer not to answer

23. In general, how many servings of fruits do you have in a day?

*(One serving = 1 medium apple, 1 cup canned fruit or 1 cup chopped fresh fruit. Includes fresh, frozen and canned fruit)*

- 0
- 1
- 2
- 3
- 4
- 5 or more
- Prefer not to answer

24. In general, how many minutes of moderate to vigorous physical activity do you participate in each week?

*Moderate to vigorous activity is an activity which makes you breathe harder such as walking, vacuuming, or yoga.*

- 75 minutes or less per week
- Between 75 to 150 minutes per week
- 150 minutes or more per week
- Prefer not to answer

25. In the last month, how often have you felt difficulties were piling up so high that you could not overcome them?
    - Never
    - Almost Never
    - Sometimes
    - Fairly Often
    - Very Often
    - Prefer not to answer
26. As an adult, how many times have you intentionally lost at least 2% of your body weight, and then gained it back?

- 0
- 1
- 2
- 3
- 4
- 5 or more
- Prefer not to answer

27. Have you been discriminated against because of your weight?

Discrimination could occur in the form of institutionalized biases (eg, in employment, healthcare, and education) and daily interpersonal biases (eg being called names).

- Yes
- No
- Prefer not to answer
