# Supplementary material for: Association Between Behavioral Phenotypes and Paid Subscription and Renewal Among New mHealth App Users: Six-Month Prospective Cohort Study
Source: J Med Internet Res. 2026 Sep 10;28:e93691. doi: 10.2196/93691 (PMC13561440; doi:10.2196/93691)
Supplement: Supplementary file 3 [file jmir-v28-e93691-s003.docx]

Survey item information

**Table S2.** Survey items, supporting references, and COM-B domain alignment.

| **Survey item** | **Association with digital health engagement? [Reference]** | **COM-B alignment** |
| --- | --- | --- |
| #1. What year were you born? | [1] | - Psychological capability - Physical capability |
| #2. What is your gender identity? | [1] | - Physical capability - Social opportunity - Physical opportunity |
| #3.What is/are your ethnic origins or ancestry? Select all geographic areas from which your ancestors first originated. | [2] | - Psychological capability - Social opportunity - Reflective motivation |
| #4. How would you identify yourself in terms of race? Select all groups that apply to you. | [2] | - Psychological capability - Social opportunity - Reflective motivation |
| #5. Are you Hispanic or  Latino/a/x | [3] | - Psychological capability - Social opportunity - Reflective motivation |
| #6. Which of the following best describes the place where you now live? | [4] | - Physical opportunity - Reflective motivation |
| #7. What was your total household income before taxes last year (in your country’s currency)? | [1] | - Physical capability - Reflective motivation |

| #9. Do you have any of the following chronic illnesses or conditions? These are conditions diagnosed by a health professional that are expected to last, or have already lasted, 6 months or more. | [5] | - Physical capability - Reflective motivation |
| --- | --- | --- |
| #10. How confident are you that you can be physically active even if you have to try several times until it works? | [6] | - Psychological capability - Physical capability - Reflective motivation |
| #11. How confident are you that you can stick to eating healthy foods even if you have to try several times until it works? | [6] | - Physical capability - Psychological capability - Reflective motivation |
| #12. How confident are you that you can lose weight even if you have to try several times until it works? | [6] | - Physical capability - Psychological capability - Reflective motivation |
| #13. How do you see yourself? Are you generally a person who is fully prepared to take risks, or do you try to avoid taking risks? | [7] | - Physical opportunity - Reflective motivation |
| “#14. I see myself as dependable and selfdisciplined” | [8] | - Reflective motivation |
| #15. “I see myself as open to new experiences” | [8] | - Reflective motivation |
| #16. “I believe that health and fitness apps are useful in my daily life” | [9] | - Reflective motivation |
| #17. “I have the knowledge necessary to use health and fitness apps” | [9] | - Physical opportunity |

| #18. “I find health and fitness apps easy to use” | [9] | - Physical capability - Physical opportunity - Reflective motivation |
| --- | --- | --- |
| #19. “People whose opinion that I value prefer I use health and fitness apps” | [9] | - Social opportunity |
| #20. “App features that make healthy living (e.g., moving more, eating better) more joyful are important to me” | [9] | - Reflective motivation |
| #21. “App features that educate me about how to  exercise best are important to me” | [9] | - Psychological capability |
| #22. In general, how many servings of vegetables do you have in a day? | [10] | - Psychological capability |
| #23. In general, how many servings of fruits do you have in a day? | [10] | - Psychological capability |
| #24. In general, how many minutes of moderate to vigorous physical activity do you participate in each week? | [3] | - Psychological capability - Reflective motivation |
| #25. In the last month, how often have you felt difficulties were piling up so high that you could not overcome them? | [11] | - Reflective motivation |
| #26. As an adult, how many times have you  intentionally lost at least 2% of your body weight, and then gained it back? | [12] | - Physical capability - Reflective motivation |
| #27. Have you been discriminated against because of your weight? | [13] | - Physical capability |

***Note:*** COM-B= Capability, Opportunity, Motivation, Behaviour, NHS=National Health Service, U.S.=United States

**References**

1.	Patel MS, Foschini L, Kurtzman GW, Zhu J, Wang W, Rareshide CAL, Zbikowski SM. Using Wearable Devices and Smartphones to Track Physical Activity: Initial Activation, Sustained Use, and Step Counts Across Sociodemographic Characteristics in a National Sample. Ann Intern Med 2017 Nov 21;167(10):755–757. PMID:28973116

2.	Chen Y, Kruahong S, Elias S, Turkson-Ocran R-A, Commodore-Mensah Y, Koirala B, Himmelfarb CRD. Racial Disparities in Shared Decision-Making and the Use of mHealth Technology Among Adults With Hypertension in the 2017-2020 Health Information National Trends Survey: Cross-Sectional Study in the United States. J Med Internet Res 2023 Sept 13;25:e47566. PMID:37703088

3.	Fendrich SJ, Balachandran M, Patel MS. Association between behavioral phenotypes and sustained use of smartphones and wearable devices to remotely monitor physical activity. Sci Rep Nature Publishing Group; 2021 Nov 2;11(1):21501. doi: 10.1038/s41598-021-01021-y

4.	Mahmood A, Kedia S, Wyant DK, Ahn S, Bhuyan SS. Use of mobile health applications for health-promoting behavior among individuals with chronic medical conditions. Digit Health 2019;5:2055207619882181. PMID:31656632

5.	Lee J-A, Choi M, Lee SA, Jiang N. Effective behavioral intervention strategies using mobile health applications for chronic disease management: a systematic review. BMC Med Inform Decis Mak 2018 Feb 20;18(1):12. PMID:29458358

6.	Balapour A, Reychav I, Sabherwal R, Azuri J. Mobile technology identity and self-efficacy: Implications for the adoption of clinically supported mobile health apps. Int J Inf Manag 2019 Dec 1;49:58–68. doi: 10.1016/j.ijinfomgt.2019.03.005

7.	Klaver NS, van de Klundert J, van den Broek RJGM, Askari M. Relationship Between Perceived Risks of Using mHealth Applications and the Intention to Use Them Among Older Adults in the Netherlands: Cross-sectional Study. JMIR MHealth UHealth 2021 Aug 30;9(8):e26845. PMID:34459745

8.	Stieger M, Robinson SA, Bisson AN, Lachman ME. The Relationship of Personality and Behavior Change in a Physical Activity Intervention: The Role of Conscientiousness and Healthy Neuroticism. Personal Individ Differ 2020 Nov 1;166:110224. PMID:32831447

9.	Yang Y, Koenigstorfer J. Determinants of Fitness App Usage and Moderating Impacts of Education-, Motivation-, and Gamification-Related App Features on Physical Activity Intentions: Cross-sectional Survey Study. J Med Internet Res 2021 July 13;23(7):e26063. PMID:34255656

10.	West JH, Belvedere LM, Andreasen R, Frandsen C, Hall PC, Crookston BT. Controlling Your “App”etite: How Diet and Nutrition-Related Mobile Apps Lead to Behavior Change. JMIR MHealth UHealth 2017 July 10;5(7):e95. PMID:28694241

11.	Kowalski L, Finnes A, Koch S, Bujacz A. User engagement with organizational mHealth stress management intervention – A mixed methods study. Internet Interv 2024 Mar 1;35:100704. doi: 10.1016/j.invent.2023.100704

12.	Macridis S, Johnston N, Johnson S, Vallance JK. Consumer physical activity tracking device ownership and use among a population-based sample of adults. PLOS ONE Public Library of Science; 2018 Jan 2;13(1):e0189298. doi: 10.1371/journal.pone.0189298

13.	Willis HA, Neblett EW. Developing culturally-adapted mobile mental health interventions: a mixed methods approach. mHealth 2023;9:1. PMID:36760787
