# Supplementary material for: Association Between Behavioral Phenotypes and Paid Subscription and Renewal Among New mHealth App Users: Six-Month Prospective Cohort Study
Source: J Med Internet Res. 2026 Sep 10;28:e93691. doi: 10.2196/93691 (PMC13561440; doi:10.2196/93691)
Supplement: Supplementary file 4 [file jmir-v28-e93691-s004.docx]

. Indicator variable selection rationale and operational definitions for engagement and device-assessed health metrics.

A clear rationale for the inclusion of all indicator variables in latent class analyses (LCAs) is important given that observed indicators determine phenotype characteristics [1]. In this study, indicator variables across three areas were selected: online survey items, device-assessed health data, and trial period engagement data. First, a 27-item survey collected data in three domains: sociodemographic (eg, gender, income), psychological (eg, physical activity self-efficacy, conscientiousness, risk taking), and health behaviours (eg, fruit and vegetable intake, weight loss attempts). This survey was systematically developed using an evidence-based approach [2,3] which included (1) choosing indicators from pre-established survey categories in similar studies, (2) selecting survey items most likely to be associated with mobile health (mHealth) engagement, and (3) ensuring survey items with at least one aspect of the Capability, Opportunity, Motivation leading to Behavior (COM-B) model were used keeping the deposit contract-based WayBetter context in mind, particularly regarding risk-taking and weight loss. By aligning survey items with conceptual links to COM-B, a more structured interpretation of how individual characteristics (eg, age) may act as potential barriers or facilitators of mHealth engagement is possible. Additionally, linking survey items to the three COM-B elements known to generate behaviour change (ie, capability, opportunity, and motivation; [4] can help support more targeted intervention recommendations in the future (eg, a user’s psychological and physical capability can be boosted through interventions targeting self-efficacy or modeling health behaviors). Once constructed, members of the research team (n=5) completed the survey to ensure question clarity and time to complete was 5–10 minutes. An attention check question (eg, “This is an attention check question. Please select ‘purple’ as your answer”) was included to assess measurement bias as well (ie, respondent inattentiveness)*.* Second, device-assessed health data, namely body weight and average daily step count, were collected during initial app sign-up. Lastly, early engagement data (eg, app opens, time spent on app) previously shown to be important for predicting WayBetter engagement [5] were collected.

**Table** **S3.** Operational definitions of app engagement metrics and device-assessed health data.

| **Variable** | **Operational Definition** |
| --- | --- |
| Total app opens | App opens, (ie, opened for one second or longer) calculated as (1) each day of trial period and (2) total during 7 days trial period. |
| Number of games joined | Total number of health-related challenges (referred to as 'games') joined by the user each day during the 7-day trial period. |
| Total time spent on app | Total time spent (in seconds) on app total during the trial period. |
| Trackers | Users who have integrated a tracker during their trial, enabling the upload of automatic fitness date. |
| Baseline Weight | Baseline weight (pounds) at the time of account creation. |
| Baseline Step Count | Average steps per day prior to trial, calculated typically over 30 days. |

**References**

1.	Sinha P, Calfee CS, Delucchi KL. Practitioner’s Guide to Latent Class Analysis: Methodological Considerations and Common Pitfalls. Critical Care Medicine 2021 Jan;49(1):e63–e79. doi: 10.1097/CCM.0000000000004710

2.	Ponto, PhD, Aprn, Agcns-Bc, Aocns® J. Understanding and Evaluating Survey Research. JADPRO 2015 Apr 1;6(2). doi: 10.6004/jadpro.2015.6.2.9

3.	Boynton PM, Greenhalgh T. Selecting, designing, and developing your questionnaire. BMJ 2004 May 29;328(7451):1312–1315. doi: 10.1136/bmj.328.7451.1312

4.	Michie S, Richardson M, Johnston M, Abraham C, Francis J, Hardeman W, Eccles MP, Cane J, Wood CE. The Behavior Change Technique Taxonomy (v1) of 93 Hierarchically Clustered Techniques: Building an International Consensus for the Reporting of Behavior Change Interventions. ann behav med 2013 Aug;46(1):81–95. doi: 10.1007/s12160-013-9486-6

5.	Jakob R, Lepper N, Fleisch E, Kowatsch T. Predicting early user churn in a public digital weight loss intervention. Proceedings of the CHI Conference on Human Factors in Computing Systems Honolulu HI USA: ACM; 2024. p. 1–16. doi: 10.1145/3613904.3642321
