# Supplementary material for: Association Between Behavioral Phenotypes and Paid Subscription and Renewal Among New mHealth App Users: Six-Month Prospective Cohort Study
Source: J Med Internet Res. 2026 Sep 10;28:e93691. doi: 10.2196/93691 (PMC13561440; doi:10.2196/93691)
Supplement: Supplementary file 5 [file jmir-v28-e93691-s005.docx]

**5.** LCA model selection criteria and BCH procedure.

To perform LCA using Mplus software (Version 1.8.10), continuous latent class indicator variables (i.e., app opens, second per day spent on app) were converted to categorical. From there, categorical variables were organized into deciles, assigning each participant to one of 10 indicator deciles. To ensure each category had sufficient representation [1], deciles with less than 10% of the sample were combined with the next highest decile until each category met or exceeded this threshold. Categorical latent class indicators (i.e., age) were grouped together in a similar way to ensure each category had no less than 10% of the sample [1]. Indicators with insufficient variability were excluded from analyses as they were unlikely to aid in class identification [2]. Survey responses “Prefer not to answer” and “I do not know” were coded as missing data. Missing indicator values were coded as -9 and declared as missing in Mplus. These values were not imputed; rather, Mplus used available observed indicator data under maximum-likelihood missing-data estimation. Thus, participants with partial indicator data could contribute to class estimation, whereas the missing -9 responses were excluded from their individual likelihood contribution.

In this study, step 1 of the Bolck-Croon-Hagenaars (BCH) approach was conducted using 33 indicator variables. A series of models were sequentially generated, starting with a one class model and then specifying the model with one additional class at a time. Models with up to seven classes were generated, as it has been suggested that classes containing fewer than 5% of the sample, or less than 50 cases, should not be used [3]. To determine the model of best fit, several fit indices in addition to theoretical interpretability were considered [3,4]. First, statistical indices, including the adjusted bayesian information criterion (aBIC) and the akaike information criterion (AIC), which aim to balance model complexity against sample size, were evaluated. Lower levels of both aBIC and AIC indicate greater model fit, although a consistent decrease in these values is often seen in large data sets with many indicators [1]. Next, likelihood indices, including the Vuong-Lo-Mendell Rubin likelihood ratio test (VLMR) [5,6] and the bootstrap likelihood ratio test (BLRT) [7], were used to determine if adding an additional class significantly improved the model fit. These two indices provide *P* values indicating if one model is statistically better than another (p < .05) [4]. Finally, models with high entropy values, a diagnostic fit statistic which illustrates class separation, were identified. To ensure a latent class model with real world application potential, theoretical interpretability was then considered [1,8]. This involved examining classes to ensure their separation was meaningful for WayBetter (eg, classes distinguishable using commonly collected metrics). Once a model of best fit was determined, the software Mplus automatically assigned individuals to their most likely class based on maximum posterior probabilities from step 2. To determine the indicator variables driving class distinction, probability weights were associated with each indicator variable, indicating the probability a participant would be categorized into a class. Similar to previously conducted latent class analyses (LCAs) [2,9], a difference threshold of 50% and 30% between the class with the highest probability and the class with the next highest probability were used to identify indicators which best distinguished classes from each other. These were labeled stronger drivers and weaker drivers, respectively. However, it is recommended that all variables in the LCA model are retained, even those with minimal discriminatory power as these variables can be important for capturing small but meaningful nuances between groups [10]. Additionally, excluding variables based only on statistical significance may lead to an oversimplified model [10]. For this reason, the full list of indicator variables was retained in the present LCA model. Finally, in step 3, the model from step 1 was re-fit using these probabilities and class membership was regressed onto two distal outcomes: subscription and 6-month renewal. Odds ratios (ORs) were generated to compare the likelihood a user in each phenotype subscribed and renewed, using class 5 as the reference [3].

**References**

1.	Sinha P, Calfee CS, Delucchi KL. Practitioner’s Guide to Latent Class Analysis: Methodological Considerations and Common Pitfalls. Critical Care Medicine 2021 Jan;49(1):e63–e79. doi: 10.1097/CCM.0000000000004710

2.	Fendrich SJ, Balachandran M, Patel MS. Association between behavioral phenotypes and sustained use of smartphones and wearable devices to remotely monitor physical activity. Sci Rep 2021 Nov 2;11(1):21501. doi: 10.1038/s41598-021-01021-y

3.	Muthén LK, Muthén BO. How to Use a Monte Carlo Study to Decide on Sample Size and Determine Power. Structural Equation Modeling: A Multidisciplinary Journal 2002 Oct;9(4):599–620. doi: 10.1207/S15328007SEM0904_8

4.	Nylund KL, Asparouhov T, Muthén BO. Deciding on the Number of Classes in Latent Class Analysis and Growth Mixture Modeling: A Monte Carlo Simulation Study. Structural Equation Modeling: A Multidisciplinary Journal 2007 Oct 23;14(4):535–569. doi: 10.1080/10705510701575396

5.	Lo Y. Testing the number of components in a normal mixture. Biometrika 2001 Oct 1;88(3):767–778. doi: 10.1093/biomet/88.3.767

6.	Vuong QH. Likelihood Ratio Tests for Model Selection and Non-Nested Hypotheses. Econometrica 1989 Mar;57(2):307. doi: 10.2307/1912557

7.	McLachlan G, Peel D. Finite Mixture Models. 1st ed. Wiley; 2000. doi: 10.1002/0471721182ISBN:978-0-471-00626-8

8.	Kongsted A, Nielsen AM. Latent Class Analysis in health research. Journal of Physiotherapy 2017 Jan;63(1):55–58. doi: 10.1016/j.jphys.2016.05.018

9.	Chen XS, Changolkar S, Navathe AS, Linn KA, Reh G, Szwartz G, Steier D, Godby S, Balachandran M, Harrison JD, Rareshide CAL, Patel MS. Association between behavioral phenotypes and response to a physical activity intervention using gamification and social incentives: Secondary analysis of the STEP UP randomized clinical trial. Krukowski RA, editor. PLoS ONE 2020 Oct 14;15(10):e0239288. doi: 10.1371/journal.pone.0239288

10.	Collins LM, Lanza ST. Latent class and latent transition analysis: with applications in the social, behavioral, and health sciences. Hoboken, NJ: Wiley; 2010. ISBN:978-0-470-22839-5
