# Supplementary material for: Association Between Behavioral Phenotypes and Paid Subscription and Renewal Among New mHealth App Users: Six-Month Prospective Cohort Study
Source: J Med Internet Res. 2026 Sep 10;28:e93691. doi: 10.2196/93691 (PMC13561440; doi:10.2196/93691)
Supplement: Supplementary file 6 [file jmir-v28-e93691-s006.docx]

Model fit information and selection criteria for latent class models.

Table S4. Model fit information and selection criteria for latent class models 1–7.

| **Class** | **Log-likelihood** | **AIC** | **aBIC** | **VLMR-LRT** | **Entropy** | **B-LRT** | **Group No.** |
| --- | --- | --- | --- | --- | --- | --- | --- |
| Class 1 | -28780.259 | 57694.517 | 57805.976 | NA | NA | NA | 934 |
| Class 2 | -25679.842 | 51605.683 | 51810.301 | 2515.473  P= 0.00 | 0.914 | -26940.544  P=0.00 | 475, 459 |
| Class 3 | -25259.930 | 50889.860 | 51197.619 | 837.848  P= 0.00 | 0.933 | -25679.842  P=0.00 | 385, 274, 275 |
| Class 4 | -24949.588 | 50393.177 | 50804.077 | 619.223  P=0.0001 | 0.951 | -25259.930  P= 0.00 | 253, 218, 235, 228 |
| **Class** **5** | **-24715.453** | **50048.906** | **50562.946** | **467.169**  **P=0.0312** | **0.921** | **-24949.588**  **P=0.00** | **166, 235, 139, 196, 198** |
| Class 6 | -24563.203 | 49868.406 | 50485.588 | 302.489  P=0.0131 | 0.965 | -25317.782  P=0.00 | 109, 185, 203, 140, 158, 139 |
| Class 7 | -24439.377 | 49744.754 | 50465.076 | 247.070  0.2556 | 0.965 | -24563.203  P= 0.00 | 92, 171, 57, 174, 142, 160, 138 |

*Note.* NA: not applicable*.* AIC: akaike information criteria. aBIC: adjusted bayesian information criteria. VLMR-LRT: Vuong-Lo-Mendell-Rubin adjusted likelihood ratio (of *k* class model to *k*-1 class model, hence none for 1 class model). B-LTR: bootstrapped likelihood ratio test. Bold indicates the selected model.
