# Supplementary material for: Association Between Behavioral Phenotypes and Paid Subscription and Renewal Among New mHealth App Users: Six-Month Prospective Cohort Study
Source: J Med Internet Res. 2026 Sep 10;28:e93691. doi: 10.2196/93691 (PMC13561440; doi:10.2196/93691)
Supplement: Supplementary file 7 [file jmir-v28-e93691-s007.docx]

Post hoc logistic regression classification accuracy.

**Table S5.** Model classification accuracy for post hoc logistic regression.

| **Observed** | **Predicted No** | **Predicted Yes** | **Percentage Correct** |
| --- | --- | --- | --- |
| No | 339 | 44 | 88.50% |
| Yes | 236 | 84 | 26.30% |
| Overall |  |  | 60.20% |
