# Supplementary material for: Association Between Behavioral Phenotypes and Paid Subscription and Renewal Among New mHealth App Users: Six-Month Prospective Cohort Study
Source: J Med Internet Res. 2026 Sep 10;28:e93691. doi: 10.2196/93691 (PMC13561440; doi:10.2196/93691)
Supplement: Supplementary file 8 [file jmir-v28-e93691-s008.docx]

**.** Post hoc logistic regression model fit statistics.

**Table S6.** Model summary of logistic regression statistics conducted in post hoc.

| **Statistic** | **Value** |
| --- | --- |
| -2 Log Likelihood | 935.054 |
| Nagelkerke R^2^ | 0.047 |
| Cox and Snell R^2^ | 0.062 |
| Hosmer-Lemeshow p | 0.358 |
